# The role of medical equipment in the spread of nosocomial infections: a cross-sectional study in four tertiary public health facilities in Uganda

**DOI:** 10.1186/s12889-020-09662-w

**Published:** 2020-10-16

**Authors:** Robert T. Ssekitoleko, Solomon Oshabaheebwa, Ian G. Munabi, Martha S. Tusabe, C. Namayega, Beryl A. Ngabirano, Brian Matovu, Julius Mugaga, William M. Reichert, Moses L. Joloba

**Affiliations:** 1 Biomedical Engineering Unit, Department of Physiology, School of Biomedical Sciences, College of Health Sciences, Makerere University, Kampala, Uganda; 2 Department of Anatomy, School of Biomedical Sciences, College of Health Sciences, Makerere University, Kampala, Uganda; 3 Duke Pratt School of Engineering, Duke University, Durham, USA; 4 Department of Microbiology, School of Biomedical Sciences, Makerere University college of Health Sciences, Kampala, Uganda

**Keywords:** Medical equipment, Nosocomial infections, Hospital acquired infections, Low and middle-income countries, Uganda

## Abstract

**Background:**

With many medical equipment in hospitals coming in direct contact with healthcare workers, patients, technicians, cleaners and sometimes care givers, it is important to pay close attention to their capacity in harboring potentially harmful pathogens. The goal of this study was to assess the role that medical equipment may potentially play in hospital acquired infections in four public health facilities in Uganda.

**Methods:**

A cross-sectional study was conducted from December 2017 to January 2018 in four public health facilities in Uganda. Each piece of equipment from the neonatal department, imaging department or operating theatre were swabbed at three distinct points: a location in contact with the patient, a location in contact with the user, and a remote location unlikely to be contacted by either the patient or the user. The swabs were analyzed for bacterial growth using standard microbiological methods. Seventeen bacterial isolates were randomly selected and tested for susceptibility/resistance to common antibiotics. The data collected analyzed in STATA version 14.

**Results:**

A total of 192 locations on 65 equipment were swabbed, with 60.4% of these locations testing positive (116/192). Nearly nine of ten equipment (57/65) tested positive for contamination in at least one location, and two out of three equipment (67.7%) tested positive in two or more locations. Of the 116 contaminated locations 52.6% were positive for *Bacillus* Species, 14.7% were positive for coagulase negative staphylococcus, 12.9% (15/116) were positive for *E. coli,* while all other bacterial species had a pooled prevalence of 19.8%. Interestingly, 55% of the remote locations were contaminated compared to 66% of the user contacted locations and 60% of the patient contacted locations. Further, 5/17 samples were resistant to at least three of the classes of antibiotics tested including penicillin, glycylcycline, tetracycline, trimethoprim sulfamethoxazole and urinary anti-infectives.

**Conclusion:**

These results provides strong support for strengthening overall disinfection/sterilization practices around medical equipment use in public health facilities in Uganda. There’s also need for further research to make a direct link to the bacterial isolates identified and cases of infections recorded among patients in similar settings.

## Background

More than 50% of all deaths in Africa are caused by infectious diseases [1]. In Uganda, bacterial infections alone were responsible for 26% of all admissions, 23% of all mortalities and 20% of all deaths in children under the age of 5 in 2018 [2]. With high numbers of admission and long patient delays, the risk of transferring infections across patients and health workers is expected to be high. The burden of nosocomial Infections is estimated to be up to twenty times higher in low-and middle-income countries (LMICs) than the high income countries [3]. Some studies conducted in sub-Saharan Africa reported prevalence rates ranging from 7 to 28% among patients admitted [3, 4]. A study by Seni et al. in Mulago National Referral Hospital, Uganda found that about 10% of patients undergoing surgical procedures become septic [5]. High incidence of nosocomial infections consequently increases the mortality and morbidity of patients especially in vulnerable populations including pediatrics, pregnant women, surgical patients and those with chronic illnesses such as HIV/AIDS that lower their immunity and often time have frequent visits to the health facilities [6–8].

Several studies have been conducted to identify the sources of bacterial infections in hospitals. Indeed bacterial colonies have been identified at various hospital environment sites including beddings [9], stethoscopes [10], computers [11], catheters [12], and other small electronic devices and instruments used by health workers [13]. However, little research has been done on the medical equipment in sub-Saharan Africa and how they can contribute to the spread of hospital acquired infections across patients and health workers.

Globally, more than 50,000 different kinds of medical equipment are estimated to be used on patients every day in hospitals [14, 15]. Despite their importance in supporting healthcare service delivery, maintenance and appropriate management of medical equipment in LMICs remains a widely recognized challenge. Medical equipment in LMICs are poorly maintained and many often neglected [16]. Studies have shown that between 30 and 50% of the equipment in low resource countries are out of service [17, 18] . Poor medical equipment maintenance in the region is partly due to lack of trained medical engineering staff to manage and maintain the available equipment as well as the highly overwhelmed healthcare workers by patient numbers [19]. This, combined with the fact that the available equipment is shared among hundreds of patients, greatly increases the risk of spread of nosocomial infections or hospital acquired infections between health workers and patients in health facilities in low resource settings. This increased risk is associated with several interrelated factors such as equipment design, user training, user competency, facility design, water quality, sterilization and disinfectant quality, infection control policies among others [17]. These risks are magnified in LMICs due to resource limitations manifested through practices such as reusing and sharing of single use medical devices or consumables as well as poor implementation of risk management policies.

The spread of nosocomial infections is worsened by the emergence of anti-microbial resistant strains of bacterial organisms which significantly increase the mortality and morbidity of bacterial infections as well as the cost of healthcare [20–22]. Preventing the spread of nosocomial infections across patients and health workers is therefore of paramount importance towards reducing the morbidity and mortality rates in low resourced countries.

Little research has been done to investigate the role of medical equipment in the spread of nosocomial infections in low resource settings. The aim of this study therefore was to identify bacterial isolates present on surfaces of medical equipment ready to be used on patients so as to assess the role of medical equipment as agents of spread of hospital acquired infections in public health facilities in Uganda.

## Methods

### Study setting and sampling

This cross-sectional study was conducted from December 2017 to January 2018, in four public health facilities located in four geographical regions in Uganda: Gulu Regional Referral Hospital (Northern Region), with a bed capacity of 397 and serving 5 districts; Kisenyi Health Centre IV (Central Region) with a bed capacity of 50 in a very busy part of Kampala, the capital city; Mbale Regional Referral Hospital (Eastern Region) with a bed capacity of 355 and serving 9 districts; and Mbarara Regional Referral Hospital (Western Region) with a bed capacity of 600 and serving 6 districts. The health facilities were chosen to represent the four different regions in Uganda. Each of them has a catchment area with a population of over 2,000,000 [Uganda MoH]. The study included all equipment that were in good working conditions in the Operation Theatre, Imaging Department and Neonatal Department. The study focused on these three departments because they employ the majority of equipment and are common across all of the chosen health facilities. All participants and hospital administrators gave written consent to participate in this study.

The different equipment and points of contact used in this study were identified with the help of unit heads as well as users and cleaners. The medical equipment employed in this study were primarily for diagnostics that had been subject to a disinfection protocol prior to use on patients. Medical equipment excluded were those not in use at the time of the study, those that were in use outside the targeted three departments, and those that had not been disinfected and considered ready to be used on patients. Medical instruments such as syringes, surgical knives, scalpels also were excluded from the study. All equipment included in the study had been disinfected within 24 h before collecting swabs.

**Fig. 1:**
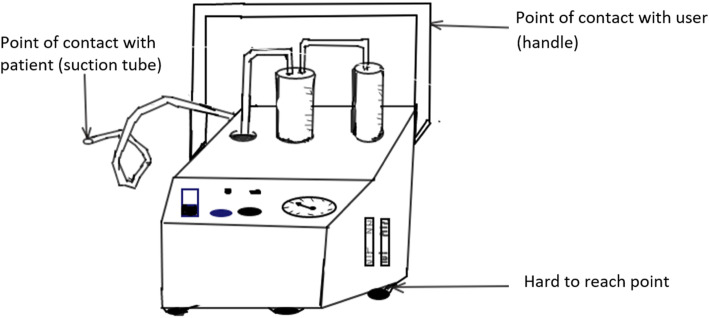
An illustration of a suction machine and the points of contact swabbed in this study (created by authors). The suction tube was swabbed as the point of contact with the patient, the handle was swabbed as the point of contact with the user, while points on the body of the equipment close to the bottom were swabbed as hard to reach areas

Samples were collected from each equipment using sterile swabs at three points: (i) the point of contact with patient, (ii) point of contact with the user and (iii) a hard to reach point which was defined as a point on the surface of equipment unlikely to be touched by neither the user nor the patient as illustrated in Fig. 1. The study however included three Overhead Phototherapy machines which did not have point of contacts with the patient thus were only swabbed at the points of contact with the user and the hard to reach points. A total of 65 pieces of equipment were swabbed with 192 total number of samples collected (62 × 3 + 3 × 2).

After analysis of samples in the case study, the single most contaminated equipment in each department at Kisenyi Health Centre IV was included in a control study. This was one ultrasound machine from the imaging department, one baby warmer in the Neonatal department and one anesthesia machine in the theatre department. This three equipment were thoroughly disinfected using 70% alcohol with great care to clean all the three points of contact targeted in the study. A 15-min time gap was allowed between disinfection and swab collection.

### Analysis of samples

The collected samples were transported to the microbiology laboratory for culture in tubes containing Amies transport medium (Biolab, Budapest, Hungary). These were packed in biohazard bags and carried in cooler boxes to the bacteriology laboratory of the Department of Microbiology at Makerere University College of Health Sciences. The samples were delivered to the laboratory within 24 h after collection and cultured upon arrival. The Samples were initially cultured on blood agar and incubated for 24 to 48 h at 37 °C in an incubator (MEMMERT) which supplies extra ingredients for accelerated bacterial growth [23, 24]. Samples were subjected to gram stain [25] and investigated using a microscope (Bestscope, Beijing, China) to differentiate the gram positive bacteria (for example *coagulase-negative staphylococci, S. aureus MRSA, and Bacillus* species) from the gram negative bacteria (for example *K. pneumoniae, E.coli, P. aeruginosa, Enterococcus* species). These colonies were further cultured on selective media using MacConkey agar and differential medial using Mannitol Salt agar to differentiate mannitol fermenting bacteria and non-mannitol fermenting bacteria for like *Staphylococcus aureus.* They were then incubated at 37 °C overnight. Thereafter, the bacterial isolates were identified using biochemical identification tests including sulfide indole motility agar for gram negative enteric bacteria (*Enterobacter* Species), Triple sugar Iron for *Pseudomonas aeruginosa*, Urea agar base for *Micrococci* species and Citrate for *Escherichia coli.* A negative control swab and a positive control swab for each of the identified bacterial isolates were included for verification.

Seventeen bacterial isolates were randomly selected and tested for susceptibility/resistance to common antibiotics. The antibiotics panel was selected based on recommendations for a standard drug susceptibility test by Clinical and Laboratory Standard Institute (CLSI) [26]. Tests were performed with the BD phoenix 100 machine (serial number: PX1302) which performs antimicrobial susceptibility testing using the broth dilution technique in which bacteria is exposed to serial dilutions of antimicrobial agents to determine whether the organisms are resistant or susceptible to the antimicrobial agent. The procedure was performed using reagents provided by the manufacturer and following the manufacturer’s instruction [27]. Briefly, the procedure was as follows; Colonies of identified micro-organisms were picked from the respective media with the tip of an applicator stick and suspended in the phoenix I.D/inoculum broth (identification broth) using 0.5 McFarland standard and vortexed for 5 s. One drop of Antimicrobial Susceptibility Testing indicator (AST) solution was then added to a labelled phoenix AST broth tube. Using a pipette, 25 μl of the bacterial suspension from the I. D tube were transferred into the AST broth tube and mixed. The AST broth inoculum was then poured into fill port on the AST side of the panel (the AST side of the combination panel contains up to 84 wells with dried antimicrobial panels and 1 growth control well). The panels selected were specific for gram negative and gram-positive bacteria as previously identified. The panels were loaded into the machine within 30 min of inoculation and incubated for sixteen hours. The results were tabulated to indicate the number of bacterial isolates resistant to each drug.

### Data analysis

The data obtained was entered into Excel and exported to STATA version 14 (College Station, Texas, USA) for analysis. Discrete variables were summarized by their means and standard deviations whereas categorical variables were presented as frequencies and percentages. Multilevel generalized linear models were used with Generalized Linear Latent and Mixed Models (GLLAMMs) for univariate analysis of factors associated with bacterial isolation from samples [28]. A three-level model was considered which included equipment level, department level and health facility level. We also specified the logit link and the binomial family in the GLLAMM model. The binomial variable for presence/absence of bacterial isolates on each sample was input as the outcome variable. The individualized associations of the health facility, department, point of contact, number of patients per equipment and number of users per equipment with the outcome was assessed in univariate analysis. A unique variable was created to test for the statistical significance of interactions between the variables that had significant associations to the outcome in the same model. The Odds ratios with respective 95% confidence intervals were used to measure the strength of associations.

## Results

As illustrated in Table 1, there were 65 medical equipment investigated in this study. These included; Anesthetic Machines (8), Baby Warmers (8), Infusion Pumps (4), Operating Tables (7), Oxygen Concentrator (13), Patient Monitors (2), Phototherapy Machines (6), Pulse Oximeter (5), Suction Machines (6), Ultrasound Machine (5), and one infant weighing Scale. Of this equipment 30.8% (20/65) were in Mbarara Regional Referral Hospital, whereas Gulu Regional Referral Hospital and Mbale Regional Referral Hospital had 26.2% (17/65) each. The lowest number of equipment was found at Kisenyi Health Centre IV, 16.7% (11/65). More than half of the equipment were from the Neonatal Department, 55.4% (35/65), whereas 38.5% (25/65) were from Surgery Theatre and the remaining 7.7% (5/65) were ultrasound machines from the Imaging Department. In general, each piece of equipment served a mean of 29 patients per week ranging from 2 to 150 and a mean of 8 users ranging from 1 to 50.

**Table 1:** Characteristics of medical equipment included in the study. RRH = Regional Referral Hospital, HC4 = Health Centre 4, SD = Standard Deviation

Characteristics		No. of pieces of equipment	Average No. of users per equipment # (SD)	Average No. of patients per week # (SD)	Disinfectant used
Equipment type	Anesthetic Machines	8 (12%)	6 (9.65)	24 (13.04)	Jik, soap, alcohol, cidex
	Baby Warmers	8 (12%)	6 (3.07)	16 (16.94)	Jik, soap, alcohol, presept, chlorhexidine
	Infusion Pumps	4 (6%)	6 (0)	35 (0)	Jik
	Operating Tables	7 (11%)	11 (17.28)	30 (15.02)	Jik, soap, alcohol, presept,
	Oxygen Concentrator	13 (20%)	7 (2.02)	19 (19.23)	Jik, soap, alcohol, chlorhexidine
	Patient Monitors	2 (3%)	18 (17.86)	18 (3.54)	Jik, soap, iodine
	Phototherapy Machines	6 (9%)	6 (2.95)	32 (23.58)	Jik, soap, alcohol, chlorhexidine
	Pulse Oximeter	5 (8%)	8 (1.41)	39 (18.30)	Jik, soap, alcohol
	Suction Machines	6 (9%)	10 (10.41)	29 (19.20)	Jik, soap, alcohol, zedex
	Ultrasound Machine	5 (8%)	5 (4.38)	80 (42.43)	Jik, soap, alcohol, saraya
	Infant weighing Scale.	1 (2%)	4	6	Jik, presept
Department	Theatre	72 (37.5%)	9 (12.48)	25 (15.02)	Jik, soap, alcohol, Presept, cidex, zedex
	Imaging	15 (7.8%)	5 (4.38)	80 (42.43)	Jik, soap, alcohol, Saraya
	Neonatal	105 (54.7%)	7 (2.22)	24 (19.41)	Jik, soap, alcohol, Presept, chlorhexidine, iodine
Health facility	Mbarara RRH	60 (31.2%)	6 (1.47)	34 (13.22)	Jik, soap, alcohol, Iodine
	Gulu RRH	51 (26.6%)	5 (2.52)	17 (21.00)	Jik, soap, alcohol, Saraya, zedex, chlorhexidine
	Kisenyi HC 4	33 (17.2%)	5 (3.30)	12 (15.81)	Jik, soap, alcohol, Presept
	Mbale RRH	48 (25.0%)	14 (13.22)	47 (30.52)	Jik, soap, alcohol, cidex

Various choices of disinfectants were used for various equipment and departments as shown in Table 1. The rationale for the choice of disinfectants was reported by the users as “we use what is available.” Some equipment such as suction machines and oxygen concentrators had autoclavable parts that get in contact with the patient. These were also swabbed as points of contact with the patient.

Nearly nine out of ten of the equipment (57/65) had bacterial isolates identified from samples taken in at least one of the three points tested, two out of three equipment (**67.7%)** tested positive in two or more locations and 27.7% (18/65) of the equipment had bacteria isolated from samples taken at all three points.

In total, 192 swabs were collected from all 65 medical equipment. Of these, 60.4% (116/192) were found colonized with a bacterial microorganism. The prevalence of bacterial isolates on samples collected from the equipment’s point of Contact with User was 66% (95% CI: 54–78%), which was significantly higher (*p*-value = 0.025) than that of samples collected from the point of contact with patient where 60% (95% CI: 47–72%) of the samples had bacterial microbes present. 55% (95% CI: 43–68%) of the samples collected from the hard to reach point where also found to have bacterial isolates present. Of the bacteria found, the most frequent isolates were *Bacillus* species 52.6% (61/116), *Coagulase negative staphylococcus* 14.7% (17/116) and *E Coli* 12.9% (15/192). The colonization of bacterial isolates also varied across departments as shown in Table 2. *Klebsiella pneumoniae* for example, which is commonly known to cause hospital acquired pneumonia, was most prevalently found at the points of contact with the patient (6.4% of the swabs from this point) and in the neonatal department (4.8% of the swabs taken from this department) which represents a potential risk to babies hospitalized in these wards.

**Table 2:** Prevalence of Bacterial Isolates identified in samples collected from the three points-of-contact for the medical equipment tested and the departments in which they were used. n represents total number of swabs in category

	Point of Contact			Department			
Bacterial microorganism	Hard to Reach (***n*** = 65)	User (n = 65)	Patient (***n*** = 62)	Neonatal (***n*** = 105)	Theatre (***n*** = 72)	Imaging (***n*** = 15)	Frequency of Isolated Bacteria (***n*** = 116)
No Bacterial growth	29 (44.6%)	22 (33.8%)	25 (40.3%)	28 (26.7%)	42 (58.3%)	6 (40%)	
**Gram negative species**							
*Escherichia coli*	3 (4.6%)	7 (10.8%)	5 (8.0%)	11 (10.5%)	4 (5.6%)		15 (12.9%)
*Klebsiella pneumoniae*	1 (1.5%)	2 (3.1%)	4 (6.4%)	5 (4.8%)	1 (1.4%)	1 (6.7%)	7 (6.0%)
*Enterobacter* species	2 (3.1%)	5 (7.7%)		6 (5.7%)	1 (1.4%)		7 (6.0%)
*P. aeruginosa*	2 (3.1%)		1 (1.6%)	2 (1.9%)	1 (1.4%)		3 (2.6%)
**Gram positive species**							
*Bacillus* species	20 (30.8%)	23 (35.4%)	18 (29.0%)	35 (33.3%)	20 (27.8%)	6 (40%)	61 (52.6%)
*Coagulase neg. Staph.*	8 (12.3%)	4 (6.2%)	5 (8.0%)	13 (12.4%)	2 (2.8%)	2 (13.3%)	17 (14.7%)
*Staphylococcus aureus*		1 (1.5%)	2 (3.2%)	2 (1.9%)	1 (1.4%)		3 (2.6%)
*Micrococcus* species		1 (1.5%)	1 (1.6%)	2 (1.9%)			2 (1.7%)
*Enterococcus* species			1 (1.6%)	1 (0.9%)			1 (0.9%)

In univariate analysis, presence of bacteria isolated from samples was associated with the categorical variables; health facility, department and point of contact from which the sample was collected. Results shown in Table 3 indicate that swabs taken from equipment in various health facilities and departments had significantly variant odds of testing positive for bacteria. Swabs from the neonatal department were more than 4 times more likely to have bacteria than swabs from the imaging department (*p*-value < 0.001). The percentage of samples that were found to have bacterial isolates from each department were: 73% (77/105) in the Neonatal Department, 60% (9/15) in the Imaging Department and 42% (30/72) in the Surgery Theatre Department. This also represents the level of risk to patients and health workers in each department. Further analysis to determine interaction between variables indicated that the interactions between the variables; health facility and department were not statistically significant (*p* value = 0.377).

**Table 3:** Association of the variables of health facility, department and point of contact with presence or absence of bacterial isolates in univariate analysis. OR is Odds ratio, CI is confidence interval, n represents total number of swabs in category

Variable		Frequency (***n*** = 192)	Bacteria Isolated (n = 116)	No Bacteria isolated (***n*** = 76)	OR (95% CI)	***P***-Value
Health Facility	Mbarara RRH	60 (31.2%)	25 (21.5%)	35 (46.0%)	1	
	Gulu RRH	51 (26.6%)	32 (27.6%)	19 (25.0%)	2.52 (1.03–6.18)	0.043
	Kisenyi HC 4	33 (17.2%)	21 (18.1%)	12 (15.8%)	2.63 (0.95–7.28)	0.063
	Mbale RRH	48 (25.0%)	38 (32.8%)	10 (13.2%)	6.07 (2.18–16.87)	0.001
Department	Theatre	72 (37.5%)	30 (25.9%)	42 (55.3%)	1	
	Imaging	15 (7.8%)	9 (7.8%)	6 (7.9%)	2.21 (0.60–8.12)	0.231
	Neonatal	105 (54.7%)	77 (66.4%)	28 (36.8%)	4.25 (1.98–9.13)	< 0.001
Point of Contact	Hard to Reach	65 (33.9%)	36 (31.0%)	29 (38.2%)	1	
	With Patient	62 (32.2%)	37 (31.9%)	25 (32.9%)	1.26 (0.58–2.72)	0.556
	With User	65 (33.9%)	43 (37.1%)	22 (28.9%)	1.70 (0.79–3.70)	0.177
No. of patients per equipment					0.99 (0.98–1.01)	0.940
No of users per equipment					1.01 (0.97–1.05)	0.642

All the 17 samples of bacteria species tested for susceptibility to antibiotics were resistant to at least four different kinds of drugs. Table 4 shows the number of bacteria colonies resistant to the various antibiotics included in the susceptibility test. All the bacterial colonies tested were resistant to Ampicillin (Penicillin class), Cephalothin (Cephalosporins class), Cefuroxime (Cephalosporins class) and Trimethoprim sulfamethoxazole class of antibiotics. Further, 5/17 samples were resistant to at least three of the classes of antibiotics tested including penicillin, glycylcycline, tetracycline, trimethoprim sulfamethoxazole and urinary anti-infectives.

**Table 4:** This table shows the number of bacterial isolates that are resistant to the various antibiotics tested against. NA implies that the bacterial species were not tested for susceptibility to that particular antibiotics

	Bacteria isolates (*n* = 17)					
Antibiotics	Acinetobacter baumannii (n = 1)	*Enterobacter cloacae* (n = 1)	Escherichia coli (n = 6)	Klebsiella pneumoniae (*n* = 4)	*Pseudomonas aeruginosa* (*n* = 2)	Staphylococcus aureus (*n* = 3)
Amoxicillin-clavulanic acid	1	1	1	3	2	2
Ampicillin	1	1	6	4	2	3
Ceftazidime	0	1	0	1	1	NA
Cephalothin	1	1	6	4	2	NA
Ceftriaxone	0	1	0	1	2	NA
Cefuroxime	1	1	6	4	2	NA
Nitrofurantoin	0	1	0	0	2	0
Cefoxitin	0	0	0	0	0	2
Mupirocin	NA	NA	NA	NA	NA	3
Oxacillin	NA	NA	NA	NA	NA	2
Penicillin G	NA	NA	NA	NA	NA	3
Trimethoprim sulfamethoxazole	1	1	6	4	2	3
Tetracycline	NA	NA	NA	NA	NA	3
Tigecycline	1	1	0	0	1	NA
Piperacillin Tazobactam	1	0	0	0	1	NA

9 samples were collected in the control study at Kisenyi Health Centre IV, of these 6/9 samples had no bacterial growth, 2/9 tested positive for bacillus species and 1/9 samples has *E coli*. Of the three samples that tested positive for contamination, one (1) was from the point of contact with the patient and the other two (2) were from the hard to reach point.

## Discussion

The number of equipment found in the four hospitals varied due to the level of each facility in the Ugandan healthcare system. Kisenyi Health center is at level four thus has less equipment than Mbarara, Gulu and Mbale Regional Referral Hospitals which are at level six. Mbarara has the biggest regional referral hospital in the country and therefore had 31% of the equipment included in the study. Similarly, the variation in the number of equipment across departments was dependent on the number of patients and services offered in the departments. For instance, the imaging department in some cases might be limited to one ultrasound machine serving up to 150 patients per week whereas the neonatal department would have several equipment with each serving one in-patient continuously over long periods of time. The phototherapy machines used to treat babies with jaundice, for example, only served 2 babies in a week.

The study showed that 88% of the medical equipment were colonized by various bacterial isolates. This is within the range of other similar studies on contamination of equipment and devices in hospitals which reported colonization rates ranging from 72 to 95% [10, 11, 13, 29]. The study found that significantly more samples collected from the neonatal department (66.4%) were colonized by bacteria than in operation theatre (25.9%). The significance of pathogens in the neonatal department was investigated by a study in Vietnamese pediatric hospitals which found a prevalence of 33% among pediatric patients [30], although studies investigating nosocomial infections among patients in sub-Saharan Africa show that surgical site infections are the most prevalent [3]. This is attributed to open surgical wounds which increase the risk of patients contracting bloodstream infections [31].

In the control study, 33.3% (3/9) of the samples still had bacteria despite being disinfected with 70% alcohol which is one of the most frequently used disinfectant against infections on hospital surfaces especially in low resourced health facilities [32]. This study showed that not all bacteria is destroyed by 70% alcohol even when properly used, which is similar to previous studies on the effectiveness of 70% alcohol in disinfecting surfaces in hospitals that showed that 36.9% of surfaces previously disinfected with alcohol tested positive for bacterial isolates [33]. However, the prevalence of infections in the control study is much lower than in the case study where 60.4% of the samples were infected which shows a lapse in the disinfection of these materials.

The isolated bacterial organisms in the case study were *Bacillus* species*, Coagulase negative staphylococcus, E. coli, Klebsiella pneumoniae, Enterobacter* species*, Pseudomonas aeruginosa, Staphylococcus aureus, Micrococcus* species and *Enterococcus* species in that order of prevalence (Table 2)*.* These have been isolated in other similar studies although the colonization rates differ across different study settings. A case study by Seni et al. in Mulago National Referral Hospital, Uganda concluded that *Staphylococcus aureus* were predominantly circulating in all the surgical wards [34]. Another study investigating the prevalence of bacterial isolates on hospital surface in Nigeria [35] found the following isolates with prevalence: *Escherichia coli (34.4%)*, *Klebsiella* species*. (21.9%), Pseudomonas* species*. (15.6%), Staphylococcus* species*. (12.5%)*, *Proteus* species (9.4%). Another similar study reported that the most common pathogen was *Enterobacter cloacae* followed by *E. coli*, *Staphylococcus aureus* and *P. aeruginosa* [36]. Other studies have identified; *Micrococcus* species*, Enterococcus* Species*, Acinetobacter* species., enterococci, coagulase-negative staphylococci, *Bacillus* species, *Legionella*, *Proteus mirabilis*, and *Serratia marcescens* [37, 38].

### Correlation to burden of infectious diseases in Uganda

The leading bacterial infections in Uganda are Pneumonia, acute diarrhoea, septicaemia, urinary tract infections, bacterial respiratory infections and bacterial meningitis. Pneumonia accounted for 9% of 1.5 million admissions recorded in 2018 and is the one the biggest killer for all ages in Uganda, second to malaria [2].. *E. coli, Klebsiella pneumoniae, Pseudomonas aeruginosa* and *Staphylococcus aureus* are common causes of hospital acquired pneumonia. In our study, these bacteria were most prevalent in samples collected from in the neonatal ward with *E-coli* being the most prevalent at 10.5% (Table 2), which illustrates a significant risk for children in admitted in these hospitals to contract hospital acquired pneumonia from the equipment.

Acute diarrhoea was the second most prevalent infectious diseases among admitted patients in Uganda with more than 72,000 reported causes of hospital admission in 2018. It has been shown that 90% of the cases of acute diarrhea are caused by microbial infections including viruses, bacteria and protozoans [39]. The bacterial causing agents of acute diarrhea that were also isolated in this study include toxin secreting species of *E-coli,* and *Staphylococcus aureus* have been previously found to cause acute diarrhea [39, 40]*..* These were also isolated on equipment in this study especially in the neonatal department (Table 2).

Septicaemia is also a common burden especially to post surgery patients, pregnant mothers and neonates. Septicemia Blood samples cultured by Kajumbura, 2014 at Makerere University College of Health Sciences yielded 187 bacterial isolates including 27% *coagulase negative staphylococci*, 18% *Staphylococcus aureus*, 10% *Klebsiella* species, 8% E-coli, 8% *Enterobacter* species, 6% *Enterococcus* species, 3% *Pseudomonas s*pecies [41]. *Bacillus* species can also cause septicemia especially in patients who are immune-compromised due to HIV infection [42]. HIV is common in Uganda with more than 2 million people estimated to be living with HIV according to the ministry of Health [2].

Urinary Tract infections (UTIs) are especially common in women due to the proximity of their urethra and anal orifices. In 2018, more than 1.7 million cases of UTIs were reported in the OPD section and accounted for 50,000 admissions in Uganda Hospitals [2]. The leading cause of UTIs is *E-coli* accounting for 80% of the cases [43, 44]. *Klebsiella* species have also been reported to cause UTIs [45]. With prevalence rates of 12 and 6% for *Escherichia coli* and *Klebsiella* pneumonia respectively, Medical equipment pose a significant threat to the spread of UTI infections among women in Ugandan Hospitals.

Although upper respiratory tract infections are the most commonly reported infections in Ugandan hospitals with prevalence of 27% in OPD attendances, majority (85%) are caused by viruses such as rhinovirus, coronavirus, respiratory syncytial virus and the parainfluenza viruses [46]. Nonetheless, up to 15% of upper respiratory infections have been reported to be caused by bacterial species including *S. pyogenes, H. influenza, Corynebacterium diphtheria, L. pneumophila, Pseudomonas aeruginosa* and *Neisseria gonorrhoeae* [47]. *Pseudomonas aeruginosa* was isolated in 2.6% of the cases identified especially in the neonatal ward (Table 2).

Bacterial meningitis is relatively uncommon in Uganda hospitals but has a relatively high death toll with about 500 annual deaths [2]. Bacterial meningitis in pediatrics is commonly caused by *Staphylococci*, *Streptococci* and gram negative *bacilli*, while in adults, is commonly caused by *S. pneumoniae* and *M. tuberculosis* and *Cryptococcus neoformans* [48]. The presence of *Streptococcus* and *Bacillus* species on equipment in the neonatal ward therefore poses a level of risk for hospital acquired bacterial meningitis among patients in these wards (Table 2).

The AMR test conducted shows that all the species tested were resistant to at least four variant strains of antibiotics and about 30% were resistant to at least three classes of antibiotics. A previous study by Sani et al., 2013 reported 78% multi drug resistance (MDR) from samples collected in Mulago national referral hospital, Uganda [5]. While, there was discrepancies likely due to the small sample space in our study, these results indicate that equipment shared among patients are a serious threat to the spread of Multi Drug Resistant strains of bacteria.

Generally, for all equipment, significantly more bacteria were isolated from the points of contact with Users than any other point on the equipment. This can be explained by the reported inappropriate use of gloves among health workers such as failure to change gloves between patients, touching multiple surfaces and patients with gloves and failure to perform hand hygiene after use [49]. The results are also probably because the healthcare workers generally use their hands more than the patients to touch the equipment, patients, medicine containers and other hospital surfaces whereas the patients on get in contact with the equipment as guided by the health workers. Indeed, approaches to strengthening medical equipment infection control practices in health facilities in low resource settings must focus on the health workers as key actors in the spread of nosocomial infections.

The significance difference in colonization rates between hospitals and departments points towards differences in infection control practices across the departments and health facilities. The theatre for example is known to have stringent rules on protective wear, restricted access which contribute to the observed significantly lower colonization rate. The neonatal department on the other hand have patients and their care takers moving about the department which increases the colonization and spread of bacterial microorganisms as seen in Table 3.

We also noted high variability in the choice of disinfectants for similar equipment even in the same health facility. This was reportedly due to variations in procurement options although it was not clear why these procurement decisions were made. There was generally a lack of uniform infection control protocols across departments and health facilities sampled which could also contribute to the high bacterial colonization rates. Previous studies have reported that decontamination of medical equipment with 70% alcohol can reduce the infection rates on medical equipment surfaces by more than 80% [50], however, low compliance to established infection control practices in health facilities in low resource settings is a major limitation to proper infection control driven by complacency among health workers and limited supply of infection control supplies when working with medical devices [51].

### Limitations

Given the small dataset available in this study, it was difficult to perform elaborate multivariate or multivariable logistic regression and account for the role of confounders. This study did not perform quantitative analysis of the bacterial colonies found which could have been useful in comparing various methods of disinfection used. This was because the laboratory used did not have any quantitative detection tools such as Rodac plates at the time of the study. Although internal controls are generally not as strong as external controls, the control study conducted in this study fulfilled its objective which was to highlight that using recommended infection control practices reduces the number of bacterial colonies on the medical equipment.

## Conclusions

Understanding the role Medical equipment plays in the spread of hospital acquired infections is extremely important in public health management since patients and health workers come into contact with this equipment very frequently. Nearly 9 of every 10 pieces of medical equipment tested in this study were positive for bacterial microorganisms which are associated with common bacterial infections in Uganda, and 2 of every 3 pieces of equipment were contaminated at two or more locations. While the user and patient contacted locations showed the highest percentage of contamination (60 and 66%, respectively), the observation that over half of the remote locations were also contaminated (55%) suggests that this equipment were not properly disinfected equipment. Further, the bacterial organisms identified were resistant to multiple antibiotics which indicates a greater risk of spread of MDR bacterial strains in the community. These results provide strong support for strengthening overall disinfection/sterilization practices around medical equipment use in public health facilities in Uganda. There’s also need for further research to make a direct link to the bacterial isolates identified and cases of infections recorded among patients in similar settings.

## Data Availability

The datasets generated and/or analysed during the current study are not publicly available due to ethics restrictions but are available from the corresponding author on a reasonable request.

## Abbreviations

AIDS: Acquired Immunodeficiency Syndrome
AMR: Antimicrobial resistance
AST: Antimicrobial Susceptibility Testing indicator
BD: Becton, Dickinson and Company
CI: Confidence Interval
CLSI: Clinical and Laboratory Standard Institute
E-Coli: *Escherichia coli*
GLLAMMs: Generalized Linear Latent and Mixed Models
*H. influenzae*: *Haemophilus influenzae*
HC4: Health Centre 4,
HIV: Human Immunodeficiency Virus
I.D: Identification
*L. pneumophila*: *Legionella pneumophila*
LMIC: Low-and middle-income country
MDR: Multi Drug Resistance
MoH: Ministry of Health
MRSA: Methicillin-resistant *Staphylococcus aureus*
OPD: Outpatients Department
OR: Odds ratio
*P. aeruginosa*: *Pseudomonas aeruginosa*
RRH: Regional Referral Hospital,
*S. pyogenes*: *Streptococcus pyogenes*
SD: Standard Deviation
UNCST: Uganda National Council for Science and Technology
UTI: Urinary Tract infections

## References

[1] Organization WH (2018). World health statistics 2018: monitoring health for the SDGs, sustainable development goals.

[2] MoH (2018). Annual Health Sector Performance Report Financial Year 2017/2018. Edited by Health Mo.

[3] Nejad SB, Allegranzi B, Syed SB, Ellis B, Pittet D (2011). Health-care-associated infection in Africa: a systematic review. Bull World Health Organ.

[4] Mbim EN, Mboto CI, Agbo BE (2016). A review of nosocomial infections in sub-Saharan Africa. Br Microbiol Res J.

[5] Seni J, Najjuka CF, Kateete DP, Makobore P, Joloba ML, Kajumbula H, Kapesa A, Bwanga F (2013). Antimicrobial resistance in hospitalized surgical patients: a silently emerging public health concern in Uganda. BMC Res Notes.

[6] Foxman B, Brown P (2003). Epidemiology of urinary tract infections: transmission and risk factors, incidence, and costs. Infect Dis Clin N Am.

[7] Carcillo JA, Dean JM, Holubkov R, Berger J, Meert KL, Anand KJ, Zimmerman J, Newth CJ, Harrison R, Burr J (2016). Inherent risk factors for nosocomial infection in the long stay critically ill child without known baseline Immunocompromise: a post–hoc analysis of the CRISIS trial. Pediatr Infect Dis J.

[8] Fisher CA (2017). Nosocomial infection in the intensive care unit: case control comparison of trauma vs surgical vs medical patients.

[9] Creamer E, Humphreys H (2008). The contribution of beds to healthcare-associated infection: the importance of adequate decontamination. J Hosp Infect.

[10] Thapa S, Sapkota LB (2017). Bacteriological assessment of stethoscopes used by healthcare workers in a tertiary care Centre of Nepal. BMC Res Notes.

[11] Schultz M, Gill J, Zubairi S, Huber R, Gordin F (2003). Bacterial contamination of computer keyboards in a teaching hospital. Infect Control Hosp Epidemiol.

[12] Nicolle LE (2014). Catheter associated urinary tract infections. Antimicrob Resist Infect Control.

[13] Khan A, Rao A, Reyes-Sacin C, Hayakawa K, Szpunar S, Riederer K, Kaye K, Fishbain JT, Levine D (2015). Use of portable electronic devices in a hospital setting and their potential for bacterial colonization. Am J Infect Control.

[14] Organization WH (2011). Core medical equipment (No. WHO/HSS/EHT/DIM/11.03).

[15] Strauss S (2000). Strauss’ pharmacy law and examination review: CRC press.

[16] Emmerling D, Dahinten A, Malkin RA. Problems with systems of medical equipment provision: an evaluation in Honduras, Rwanda and Cambodia identifies opportunities to strengthen healthcare systems. Heal Technol. 2018;8(1-2): 129–35.

[17] Perry L, Malkin R. Effectiveness of medical equipment donations to improve health systems: how much medical equipment is broken in the developing world? Springer; 2011. 10.1007/s11517-011-0786-3 21597999

[18] Ademe BW, Tebeje B, Molla A (2016). Availability and utilization of medical devices in Jimma zone hospitals, Southwest Ethiopia: a case study. BMC Health Serv Res.

[19] Ploss B, Reichert W (2017). Part I. the emergence of degree-granting biomedical engineering programs in sub-Saharan Africa. Ann Biomed Eng.

[20] Poorabbas B, Mardaneh J, Rezaei Z, Kalani M, Pouladfar G, Alami MH, Soltani J, Shamsi-Zadeh A, Abdoli-Oskooi S, Saffar MJ (2015). Nosocomial infections: multicenter surveillance of antimicrobial resistance profile of Staphylococcus aureus and gram negative rods isolated from blood and other sterile body fluids in Iran. Iran J Microbiol.

[21] Fiore M, Maraolo AE, Gentile I, Borgia G, Leone S, Sansone P, Passavanti MB, Aurilio C, Pace MC (2017). Nosocomial spontaneous bacterial peritonitis antibiotic treatment in the era of multi-drug resistance pathogens: a systematic review. World J Gastroenterol.

[22] Dondorp AM, Limmathurotsakul D, Ashley EA (2018). What’s wrong in the control of antimicrobial resistance in critically ill patients from low-and middle-income countries?. Intensive Care Med.

[23] Stieglmeier M, Wirth R, Kminek G, Moissl-Eichinger C (2009). Cultivation of anaerobic and facultatively anaerobic bacteria from spacecraft-associated clean rooms. Appl Environ Microbiol.

[24] Brook I (2002). Aerobic and anaerobic microbiology of suppurative sialadenitis. J Med Microbiol.

[25] Kozlov A, Bean L, Hill EV, Zhao L, Li E, Wang GP (2018). Molecular identification of bacteria in intra-abdominal abscesses using deep sequencing. Open forum infectious diseases: 2018.

[26] Wayne P (2011). Performance standards for antimicrobial susceptibility testing; Twenty-first Informational Supplement. CLSI Document M100-S21, Clinical and Laboratory Standards Institute.

[27] LABORATORY PROCEDURE - BD Phoenix™ PMIC/ID Panels [https://legacy.bd.com/ds/technicalCenter/clsi/clsi-Phoenix_GramPositive_V5.15_V4.31.pdf]. Accessed 16 Aug 2020.

[28] Skrondal A, Rabe-Hesketh S. Some applications of generalized linear latent and mixed models in epidemiology: repeated measures, measurement error and multilevel modeling. Norsk Epidemiologi. 2003;13(2):5–7.

[29] Fafliora E, Bampalis VG, Lazarou N, Mantzouranis G, Anastassiou ED, Spiliopoulou I, Christofidou M (2014). Bacterial contamination of medical devices in a Greek emergency department: impact of physicians’ cleaning habits. Am J Infect Control.

[30] Le NK, Wertheim H, Vu PD, Khu DTK, Le HT, Hoang BTN, Vo VT, Lam YM, Vu DTV. Nguyen TH: High prevalence of hospital-acquired infections caused by gram-negative carbapenem resistant strains in Vietnamese pediatric ICUs: A multi-centre point prevalence survey. Medicine. 2016;95(27):807–9. 10.1097/MD.0000000000004099 PMC5058835 27399106

[31] Reddy EA, Shaw AV, Crump JA (2010). Community-acquired bloodstream infections in Africa: a systematic review and meta-analysis. Lancet Infect Dis.

[32] Boyce JM (2018). Alcohols as surface disinfectants in healthcare settings. Infect Control Hosp Epidemiol.

[33] Rutala WA, Weber DJ (2016). Disinfection and sterilization in health care facilities: an overview and current issues. Infect Dis Clin.

[34] Seni J, Bwanga F, Najjuka CF, Makobore P, Okee M, Mshana SE, Kidenya BR, Joloba ML, Kateete DP (2013). Molecular characterization of Staphylococcus aureus from patients with surgical site infections at Mulago Hospital in Kampala, Uganda. PLoS One.

[35] Ameh EA, Mshelbwala PM, Nasir AA, Lukong CS, Jabo BA, Anumah MA, Nmadu PT (2009). Surgical site infection in children: prospective analysis of the burden and risk factors in a sub-Saharan African setting. Surg Infect.

[36] Dia N, Ka R, Dieng C, Diagne R, Dia M, Fortes L, Diop B, Sow A, Sow P (2008). Prevalence of nosocomial infections in a university hospital (Dakar, Senegal). Medecine et maladies infectieuses.

[37] Atif ML, Sadaoui F, Bezzaoucha A, Kaddache CA, Boukari R, Djelato S, Boubechou N (2009). Reduction of nosocomial pneumonia using surveillance and targeted interventions in an Algerian neonatal intensive care unit. Infect Control Hosp Epidemiol.

[38] Eriksen H, Chugulu S, Kondo S, Lingaas E (2003). Surgical-site infections at Kilimanjaro Christian medical center. J Hosp Infect.

[39] Ahlquist D, Camilleri M (2001). Diarrhea and constipation. HARRISONS PRINCIPLES INTERN MED.

[40] Casburn-Jones A, Farthing M (2004). Management of infectious diarrhoea. Gut.

[41] Kajumbula H (2014). Routine findings (M. laboratory, Trans.). Microbiology Makerere University College of Health Sciences.

[42] Drobniewski FA (1993). Bacillus cereus and related species. Clin Microbiol Rev.

[43] Kaper JB, Nataro JP, Mobley HL (2004). Pathogenic escherichia coli. Nat Rev Microbiol.

[44] Kumar M, Rawat V, Singh MA, Bahugune D, Joshi S, Kumar U. Sphingomonas paucimobilis urinary tract infection in an immunecompetent patient: A case report. Int J Med Public Health. 2015;5(2):123–40.

[45] Latifpour M, Gholipour A, Damavandi MS. Prevalence of extended-spectrum beta-lactamase-producing Klebsiella pneumoniae isolates in nosocomial and community-acquired urinary tract infections. Jundishapur J Microbiol. 2016;9(3):204. 10.5812/jjm.31179 PMC4877671 27226874

[46] Adams O, Weis J, Jasinska K, Vogel M, Tenenbaum T (2015). Comparison of human metapneumovirus, respiratory syncytial virus and rhinovirus respiratory tract infections in young children admitted to hospital. J Med Virol.

[47] Durand M, Joseph M (2001). Infections of the Upper Respiratory Tact in Principles of Internal Medicine.

[48] Iriso R, Ocakacon R, Acayo J, Mawanda M, Kisayke A (2008). Bacterial meningitis following introduction of Hib conjugate vaccine in northern Uganda. Ann Trop Paediatr.

[49] Loveday H, Lynam S, Singleton J, Wilson J (2014). Clinical glove use: healthcare workers' actions and perceptions. J Hosp Infect.

[50] Schabrun S, Chipchase L (2006). Healthcare equipment as a source of nosocomial infection: a systematic review. J Hosp Infect.

[51] Bischoff WE, Reynolds TM, Sessler CN, Edmond MB, Wenzel RP (2000). Handwashing compliance by health care workers: the impact of introducing an accessible, alcohol-based hand antiseptic. Arch Intern Med.

